# Folate Deficiency Drives Metabolic Dysfunction, Oxidative Stress, and Inflammation in HK-2 Renal Tubular Cells

**DOI:** 10.3390/ijms27198846

**Published:** 2026-10-03

**Authors:** Te-I Chiu, Hsiang-Yu Liu, Bi-Fong Lin

**Affiliations:** Department of Biochemical Science and Technology, College of Life Science, National Taiwan University, Taipei 10617, Taiwanr13b22033@ntu.edu.tw (H.-Y.L.)

**Keywords:** folate, inflammation, chronic kidney disease, oxidative stress, lactate, lactate dehydrogenase

## Abstract

The growing aging population has been accompanied by an increasing prevalence of chronic kidney disease (CKD), highlighting the need to better understand modifiable dietary and metabolic factors that contribute to renal injury. Folate deficiency and excessive fructose intake have been implicated in renal inflammation; however, the underlying cellular mechanisms remain incompletely understood. In this study, we investigated the effects of folate deficiency or fructose exposure on inflammatory responses, lactate metabolism, oxidative stress, and mitochondrial function in human renal tubular epithelial HK-2 cells. Folate deficiency markedly increased the secretion of the pro-inflammatory cytokines interleukin-6 (IL-6) and monocyte chemoattractant protein-1 (MCP-1), accompanied by enhanced NF-κB activation, both in the presence and absence of lipopolysaccharide (LPS) stimulation. Notably, folate-deficient cells exhibited increased lactate dehydrogenase (LDH) activity, intracellular lactate accumulation, and lactate secretion. Exogenous lactate supplementation or modulation of lactate production using the LDH inhibitor oxamate further demonstrated that elevated lactate promoted IL-6 and transforming growth factor-β1 (TGF-β1) production, NF-κB activation, and reactive oxygen species (ROS) generation. Consistent with these findings, folate deficiency significantly reduced the oxygen consumption ratio (OCR), indicating impaired mitochondrial function and a metabolic shift away from oxidative phosphorylation toward increased lactate production. High fructose exposure also enhanced IL-6 and MCP-1 secretion, particularly following LPS stimulation, while both LPS exposure and folate deficiency further increased lactate production. Collectively, these findings identify lactate accumulation as a potential metabolic link between folate deficiency, inflammation, oxidative stress and fibrotic signaling in renal tubular HK-2 cells. This metabolic-inflammatory pathway may contribute to the initiation and progression of CKD and highlights folate status and lactate metabolism as potential targets for preventing diet-associated renal injury.

## 1. Introduction

The prevalence of chronic kidney disease (CKD) is increasing in aging societies, as aging is associated with declines in body function and size, impaired cellular repair, and dysregulation of physiological processes. A recent review reported that dietary transitions toward modern dietary patterns may contribute to the growing global burden of CKD through interconnected metabolic, inflammatory, and gut–kidney pathways [1]. Western dietary patterns, characterized by high intake of fat, simple sugars, and sodium, together with low intakes of fiber, fruits, and vegetables, and/or high consumption of ultra-processed foods, are associated with an increased risk of CKD [2]. In contrast, healthy dietary patterns are associated with a lower incidence of CKD, particularly the Mediterranean diet, which is characterized by a high daily intake of fresh fruits and vegetables [3,4].

Fresh fruits and vegetables are rich sources of essential nutrients, including vitamins such as vitamin C and vitamin A, mineral such as potassium, and dietary fiber. Dark green leafy vegetables and legumes are also important natural sources of folate, whereas light-colored vegetables and fruits contain relatively little folate [5,6]. Although enriched grain products, such as breads, pastas, and cereals are major dietary contributors because they are widely fortified with synthetic folic acid, mandatory folic acid fortification policies have not been implemented in more than 100 countries, including countries in the European Union [7]. Therefore, folate status remains a concern, particularly among individuals with poor or unbalanced diets [8,9]. Folate deficiency increases lipid accumulation and leptin production by adipocytes, which may contribute to systemic inflammation [10]. Folate deficiency or insufficiency may also exacerbate inflammation and promote renal fibrosis [11].

Persistent low-grade inflammation contributes to the progressive decline of kidney function. Unresolved renal inflammation induces cellular changes that promote the release of profibrotic signals, ultimately leading to excessive accumulation of scar tissue and fibrosis. Chronic kidney inflammation represents a sustained immune response that causes progressive damage to renal tissue. Therefore, maintaining long-term healthy dietary habits, consuming anti-inflammatory nutrients, and preventing chronic inflammation are essential strategies for reducing the risk of CKD. Recent reviews have described an anti-inflammatory diet as one characterized by the regular consumption of foods with potential anti-inflammatory properties, including fruits, vegetables, whole grains, nuts, legumes, spices, herbs, and plant-based proteins [12]. These dietary components are consistent with the characteristics of healthy dietary patterns associated with a lower incidence of CKD [2]. Several dietary factors have been reported to exert anti-inflammatory effects in our previous studies [13,14,15]. Our previous study demonstrated that anti-inflammatory dietary factors can suppress the expression of pro-inflammatory and profibrotic cytokines, including IL-6, TNF-α, MCP-1, IL-17A, and TGF-β1, thereby ameliorating renal injury [16]. The rationale for anti-inflammatory diets lies in their abilities to reduce levels of pro-inflammatory biomarkers, modulate signal pathways involved in the inflammatory cascade, and suppress the activity of inflammatory immune cells [12].

Meanwhile, poor dietary quality and unbalanced diets characterized by low vegetables intake and inadequate consumption of essential nutrients may lead to micronutrient deficiencies or insufficiencies. However, few studies have focused on the effects of nutrient deficiencies on inflammation, as suppression of immune cell activity and proliferation has traditionally been considered the primary consequence of nutrient deficiency, resulting in reduced cytokine production [17]. Several major micronutrients, including vitamins A, D, E, and C, as well as zinc and selenium, have been investigated as immunomodulators in the aging population, particularly in relation to the process of inflammaging. Folate deficiency has also been linked to impaired immune responses [18]. We previously showed that folate deficiency promotes IL-6 and MCP-1 production in adipocytes and renal homogenates from mice fed a high-fat, high-fructose diet [10,11]. However, the mechanisms underlying this pro-inflammatory effect remain unclear.

It has been established that transcription factor NF-κB promotes immune responses by regulating the expression of genes involved in inflammation. NF-κB activation can occur in most cell types and plays a key role in the liver, adipose tissue, and central nervous system in the development of inflammation-associated metabolic diseases [19]. The ratio or relative levels of phosphorylated p65 (phospho-p65) to total p65 is commonly used as a molecular readout of activation of the canonical NF-κB inflammatory and stress-signaling pathway. An increased phosph-p65/p65 ratio indicates pathway activation in response to various pathological stimuli. Renal tubular cells express numerous pro-inflammatory cytokines, chemokines, and fibrosis-related mediators and play a critical role in the response to kidney injury. In this study, we further investigate the mechanisms underlying the effects of folate deficiency on inflammatory responses in renal tubular cells, with a particular focus on metabolic alterations, oxidative stress, and mitochondrial dysfunction.

Metabolic reprogramming in renal tubular epithelial cells has emerged as an important mechanism driving renal inflammation and fibrosis. In a mouse model of ischemia–reperfusion injury, Wang et al. showed that increased expression of the glycolytic enzyme 6-phosphofructo-2-kinase/fructose-2,6-bisphosphatase 3 (PFKFB3) in proximal tubular cells promotes lactate accumulation and transcription of NF-κB-related genes through histone H4 lysine 12 lactylation (H4K12 lactylation), thereby exacerbating renal inflammation and fibrosis. Notably, urinary lactate-to-creatinine ratios and tissue lactate levels were positively correlated with the severity of renal fibrosis in patients with CKD [20]. Subsequent reviews have further implicated lactate metabolism and lactylation-mediated epigenetic regulation in tubular injury, fibrosis and the AKI-to-CKD transition [21,22]. Inhibiting of tubular glycolysis has likewise been shown to attenuate renal interstitial fibrosis through distinct mechanisms, further supporting a role for the glycolytic/lactate metabolic axis in renal fibrosis in animal models [23]. Collectively, these findings support a link between dysregulated glycolysis/lactate metabolism in renal tubular epithelial cells and NF-κB-mediated renal inflammation and fibrosis. However, the upstream stimuli investigated to date have predominately been pathological conditions, such as ischemia–reperfusion injury, ureteral obstruction, and diabetic kidney disease, whereas the role of nutrient deficiency-induced metabolic stress remains poorly understood.

Fructose-induced renal injury has been associated with activation of NF-κB signaling and impaired Nrf2 nuclear translocation, leading to enhanced inflammation and oxidative stress [24]. Consistent with these findings, we observed increased IL-6 and MCP-1 secretion following fructose treatment of HK-2 cells. However, whether altered lactate metabolism contributes to fructose-induced renal inflammation remains unknown.

Clinical evidence increasingly links folate status to CKD; for example, in the China Stroke Primary Prevention Trial (CSPPT), folic acid supplementation combined with enalapril slowed CKD progression compared with enalapril alone, while 30-year follow-up of the Coronary Artery Risk Development in Young Adults (CARDIA) cohort showed that higher dietary folate intake in young adulthood was associated with lower CKD incidence in midlife [25,26]. However, the underlying cellular mechanisms remain poorly defined. In particular, whether folate deficiency induces metabolic reprogramming—including lactate accumulation—thereby promotes tubular inflammation, oxidative stress, and fibrosis remains unknown.

Lactate-related metabolic alterations have also been associated with adverse renal outcomes. In a secondary analysis of the PREDICT trial, higher serum lactate dehydrogenase (LDH) levels were associated with increased risks of kidney disease progression, cardiovascular events, and death in patients with severe non-diabetic CKD [27]. These clinical observations provide translational support for investigating the LDH-lactate axis as a potential metabolic link between folate deficiency and renal injury.

Collectively, the current evidence links dysregulated glycolysis/lactate metabolism to NF-κB-mediated renal inflammation and fibrosis, while clinical studies associate folate status with CKD risk and progression. However, whether folate deficiency engages the LDH–lactate axis to promote tubular inflammation and metabolic dysfunction remains unknown. Here, we identify lactate metabolism as a potential mechanistic link between folate deficiency and the lactate–NF-κB inflammatory and fibrotic axis. Our findings extend the known upstream triggers of this pathway beyond ischemic and diabetic injury to nutrient deficiency, providing a potential cellular mechanism linking folate and LDH-related alterations to CKD pathogenesis.

## 2. Results

### 2.1. Folate and Inflammatory Cytokine Secretion in HK-2 Cells

**Figure 1:**
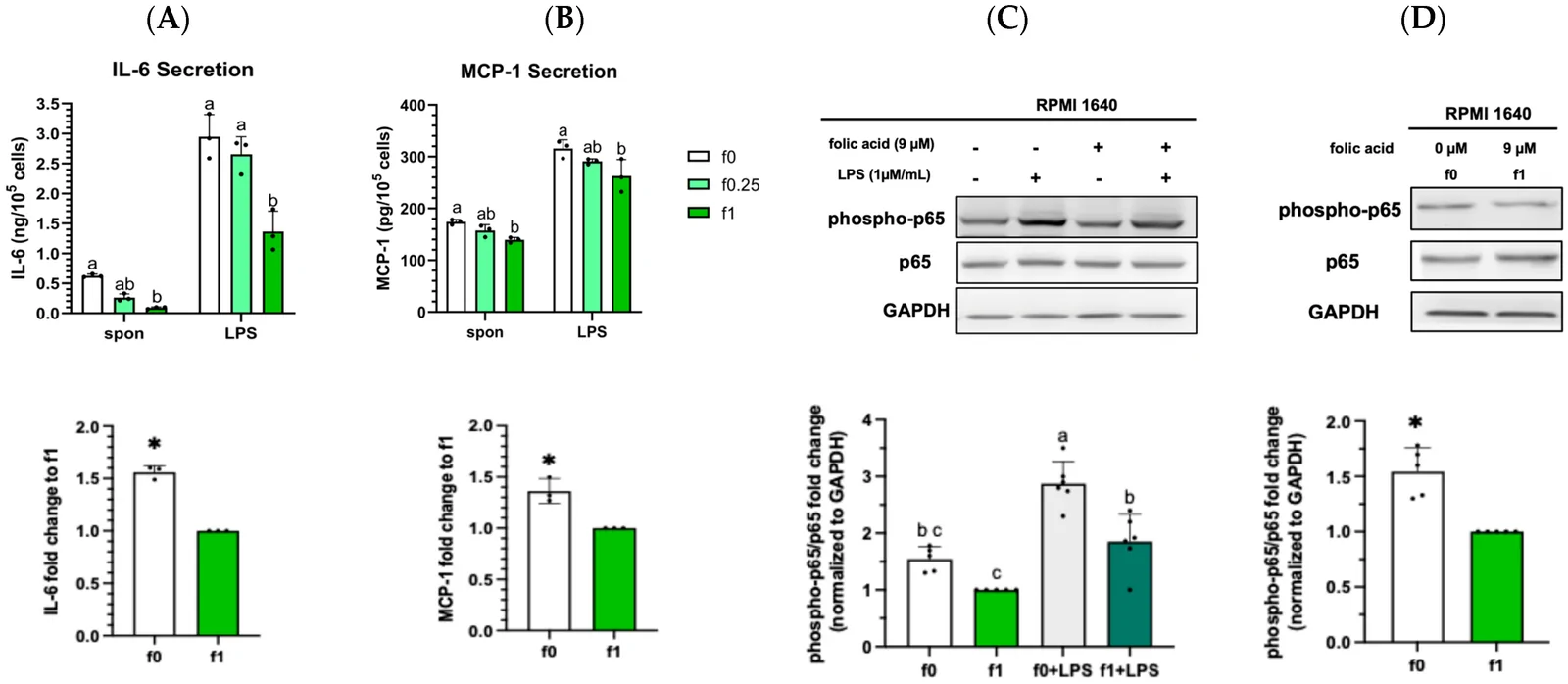
Effects of folate concentrations on pro-inflammatory secretion and NF-κB pathway activation in HK-2 cells with or without LPS stimulation. (**A**) IL-6 secretion, (**B**) MCP-1 secretion, and (**C**,**D**) the ratio of phosphorylated p65 to total p65 (phospho-p65/p65) following 24-h LPS stimulation (**C**) and in the absence of LPS stimulation (**D**). Cells were cultured in folate-deficient medium (f0 (0 mg/L folate) or f0.25 (0.25 mg/L folate)) or folate-sufficient medium (f1 (1 mg/L folate)) for 4 days. Data are presented as mean ± SD (*n* = 3–6 independent experiments). Different letters indicate significant differences among groups (*p* < 0.05; one-way ANOVA followed by Duncan’s post hoc test). * *p* < 0.05 versus the folate-sufficient culture (f1 or f1 + LPS).

To investigate whether folate deficiency promotes the secretion of pro-inflammatory cytokines, IL-6 and MCP-1 secretion by HK-2 cells was measured following 4 days of culture under folate-deficient conditions. As shown in Figure 1A,B, folate deficiency significantly increased IL-6 and MCP-1 secretion by HK-2 cells, with a dose-dependent trend, both in the absence and presence of LPS. These findings were further confirmed in the absence of LPS stimulation to determine whether folate-deficiency promotes spontaneous secretion of IL-6 and MCP-1. The data are expressed as fold changes because the basal levels of spontaneously secreted pro-inflammatory cytokines by HK-2 cells were relatively low.

We also investigated whether NF-κB activation was affected by folate status. Consistent with the increased secretion of pro-inflammatory cytokines, Figure 1C shows that folate deficiency tended to increase spontaneous NF-κB activation and significantly enhanced NF-κB activation following LPS stimulation. Figure 1D shows the fold changes in spontaneous of NF-κB signaling activation induced by folate deficiency.

### 2.2. Folate Deficiency and Lactate Production in HK-2 Cell Culture

**Figure 2:**
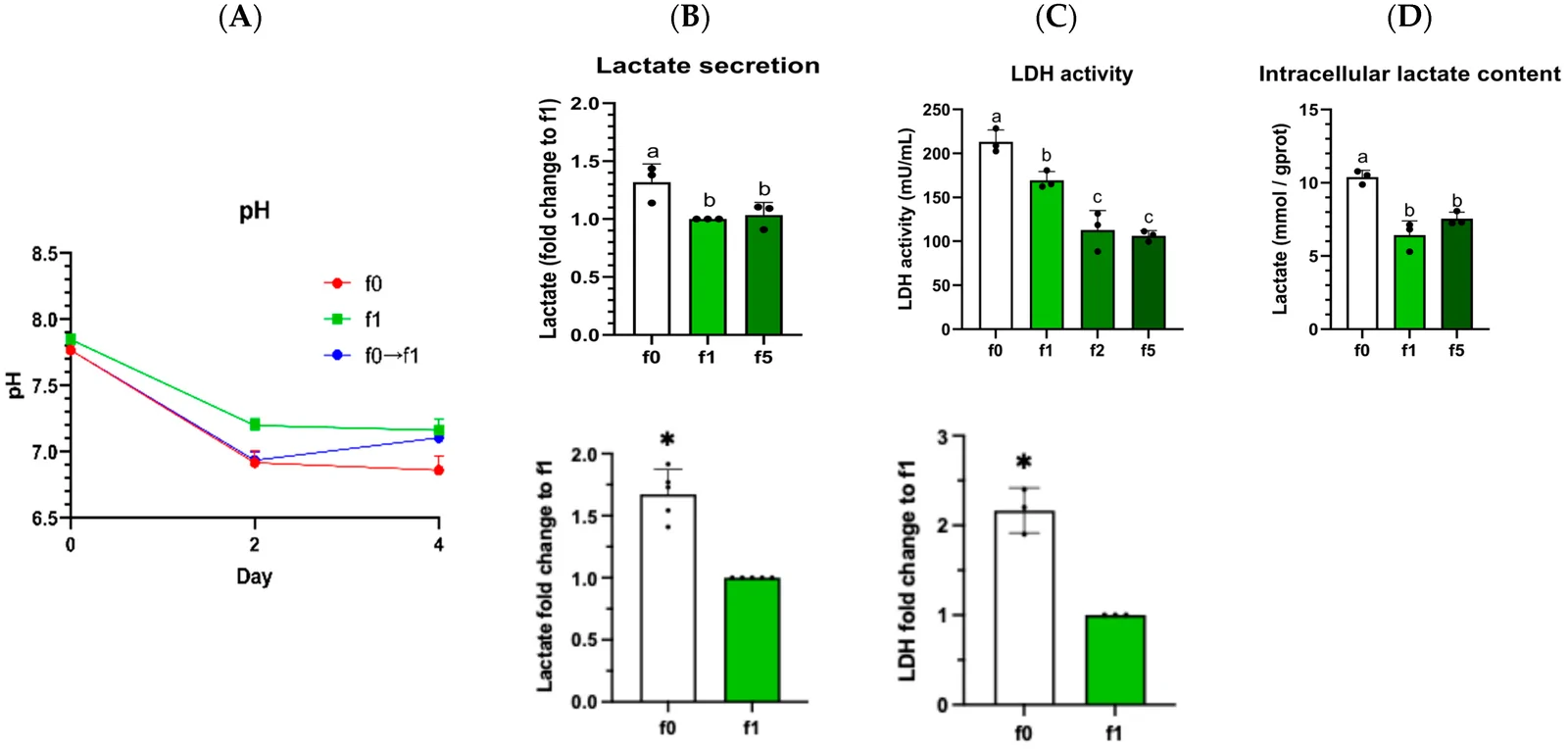
Effects of folate status on lactate production and lactate dehydrogenase activity in HK-2 cells cultured with varying folate concentrations. (**A**) Changes in culture medium pH during culture under folate deficiency (f0), folate-sufficiency (f1), or folate-replenished (f0 → f1) conditions. (**B**) Fold change in extracellular lactate levels under f0 versus f1 conditions. (**C**) Lactate dehydrogenase (LDH) activity in cells cultured with varying folate concentrations and fold change in LDH activity under f0 versus f1 conditions. (**D**) Intracellular lactate levels in cells cultured with varying folate concentrations. Cells were cultured in folate-deficient medium (f0; 0 mg/L folate), folate-sufficient medium (f1; 1 mg/L folate), or folate-supplemented medium (f2 (2 mg/L folate) or f5 (5 mg/L folate)) for 4 days. Data are presented as mean ± SD (*n* = 3–5 independent experiments). Different letters indicate significant differences among groups (*p* < 0.05; one-way ANOVA followed by Duncan’s post hoc test). * *p* < 0.05 compared with the f1 culture.

During the cell culture, the culture medium of HK-2 cells turned yellow more rapidly under folate-deficient conditions. To quantify this observation, the pH of the culture supernatants was measured after 2 days of culture. Supernatants from the folate-deficient group exhibited a greater decrease in pH than those from folate-sufficient group, whereas folate replenishment attenuated this acidification (Figure 2A). These findings suggest that folate deficiency alters the metabolic prolife of HK-2 cells and increases the accumulation of acidic metabolic products.

Given the important role of the metabolic shift from fatty acid oxidation toward lactate production in the progression of both acute and chronic kidney disease, we hypothesized that acidification of the supernatant may result from increased lactate production. To investigate this possibility, extracellular lactate levels (Figure 2B), LDH activity (Figure 2C), and intracellular lactate accumulation (Figure 2D) were measured. Folate deficiency significantly increased extracellular lactate levels, LDH activity, and intracellular lactate accumulation. Although folate supplementation did not significantly affect extracellular lactate levels or intracellular lactate accumulation, it significantly reduced LDH activity. Taken together, these findings suggest that folate deficiency enhanced LDH activity, thereby promoting intracellular lactate accumulation and extracellular lactate secretion in renal tubular cells.

### 2.3. Lactate and Inflammatory Cytokine Secretion in HK-2 Cells

**Figure 3:**
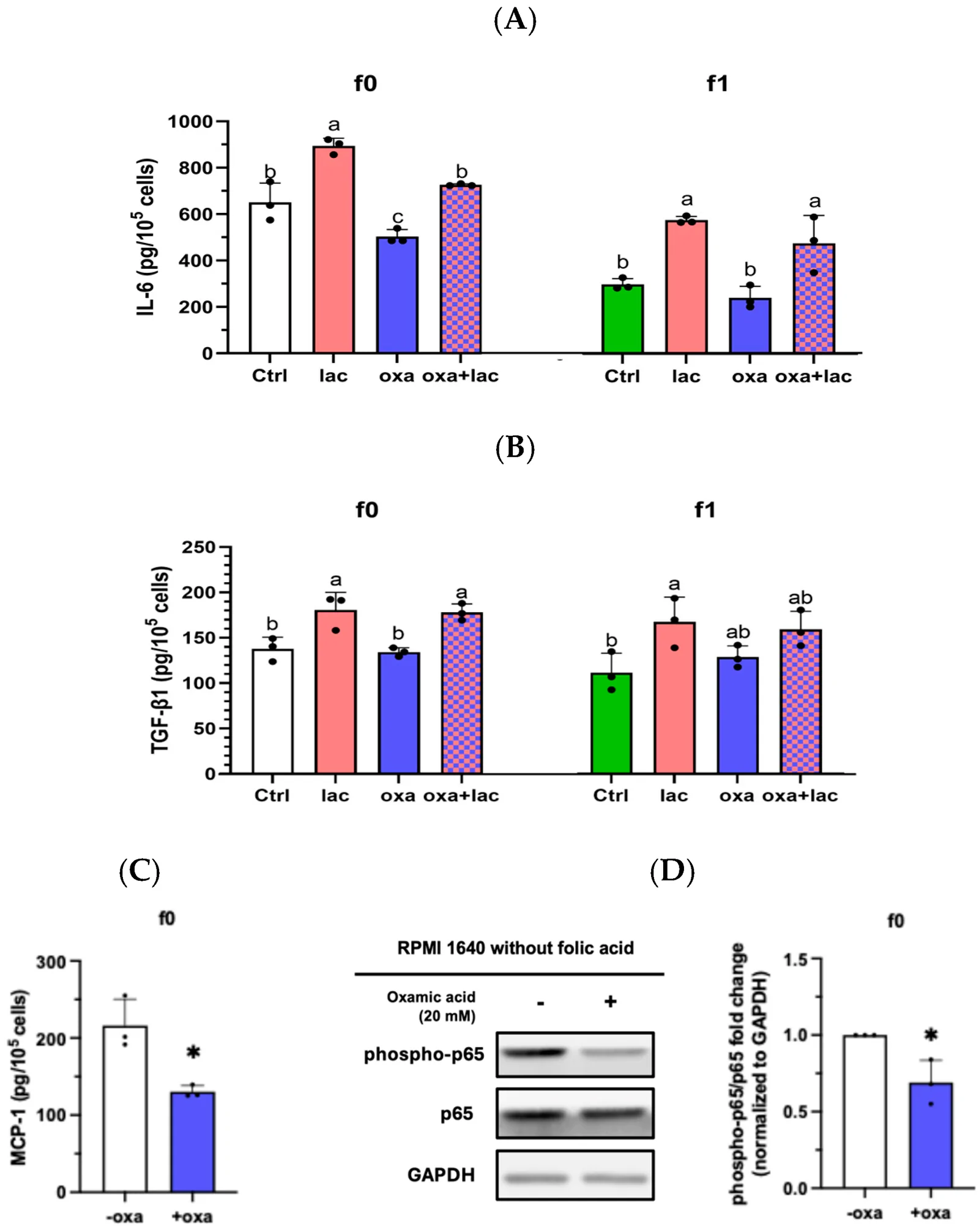
Effects of lactate supplementation and LDH inhibition on cytokine secretion and NF-κB activation in HK-2 cells. (**A**) IL-6 and (**B**) TGF-β1 secretion by HK-2 cells cultured with folate-deficient (f0) or folate-sufficient (f1) conditions, with or without lactate supplementation (10 mM) and LDH inhibition (20 mM oxamate). (**C**) MCP-1 secretion and (**D**) the phospho-p65/p65 ratio in folate-deficient HK-2 cells following lactate supplementation and/or LDH inhibition (oxamate treatment; +oxa). Cells were cultured in folate-deficient medium (f0; 0 mg/L folate) or folate-sufficient medium (f1; 1 mg/L folate) for 4 days. Data are presented as mean ± SD (*n* = 3 independent experiments). Different letters indicate significant differences among groups within the same treatment condition (*p* < 0.05; one-way ANOVA followed by Duncan’s post hoc test). * *p* < 0.05 compared with the corresponding control without oxamate (-oxa).

To investigate the potential relationship between lactate production and cytokine secretion in folate-deficient HK-2 cells, exogenous lactate and oxamate, an inhibitor of LDH, were applied to the cells. Under folate-sufficient conditions, exogeneous lactate significantly increased IL-6 and TGF-β1 secretion, regardless of whether lactate production was inhibited (Figure 3A,B), suggesting that lactate itself may contribute to the increased secretion of IL-6 and TGF-β1.

Under folate-deficient conditions, exogenous lactate similarly increased IL-6 and TGF-β1 secretion. However, these increases were attenuated by LDH inhibition. In addition, oxamate treatment reduced MCP-1 secretion and NF-κB pathway activation under folate-deficient conditions (Figure 3C,D). Taken together, these findings suggest that exogenous lactate can increase IL-6 and TGF-β1 secretion, whereas lactate itself and/or endogenous lactate production may contribute to the regulation of pro-inflammatory cytokine secretion and NF-κB activation in folate-deficient HK-2 cells.

### 2.4. Lactate and Oxidative Stress in HK-2 Cells

**Figure 4:**
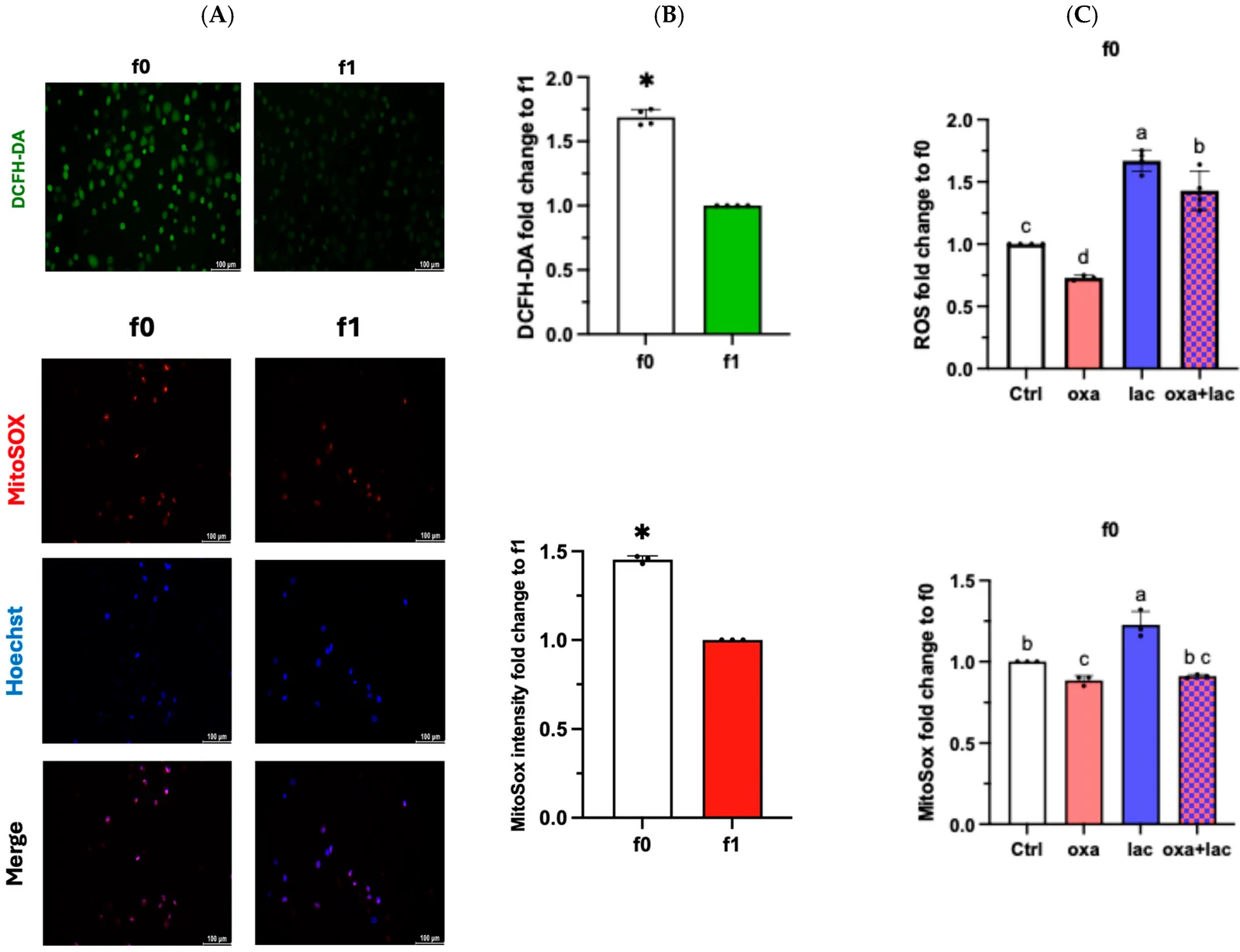
Effects of folate deficiency and lactate on oxidative stress in HK-2 cells. (**A**) Representative fluorescence images showing intracellular and mitochondrial ROS levels induced by folate deficiency, measured using the DCFH-DA and MitoSOX assays, respectively. DCFH-DA fluorescence is shown in green, MitoSOX fluorescence in red, and Hoechst nuclear staining in blue. Scale bar = 100 μm. (**B**) Quantification of ROS levels based on relative fluorescence intensity. (**C**) Quantification of ROS levels following inhibition of endogenous lactate production with the LDH inhibitor oxamate (oxa; 20 mM) or supplementation with exogenous lactate (lac; 10 mM), based on relative fluorescence intensity. Cells were cultured in folate-deficient medium (f0; 0 mg/L folate) or folate-sufficient medium (f1; 1 mg/L folate) for 4 days. Data are presented as mean ± SD (*n* = 3–4 independent experiments). * *p* < 0.05 compared with the f1 culture. Different letters indicate significant differences among groups (*p* < 0.05; one-way ANOVA followed by Duncan’s post hoc test).

As shown in Figure 4A,B, folate-deficient HK-2 cells exhibited higher levels of both cellular and mitochondrial oxidative stress. To investigate whether these effects were also associated with lactate, oxamate, an LDH inhibitor, was used to reduce endogenous lactate production. Inhibition of endogenous lactate production reduced both intracellular and mitochondrial ROS levels (Figure 4C). In contrast, exogeneous lactate supplementation increased intracellular and mitochondrial ROS levels, and these effects were attenuated by treatment with the LDH inhibitor.

### 2.5. ROS Production and Lactate Levels

**Figure 5:**
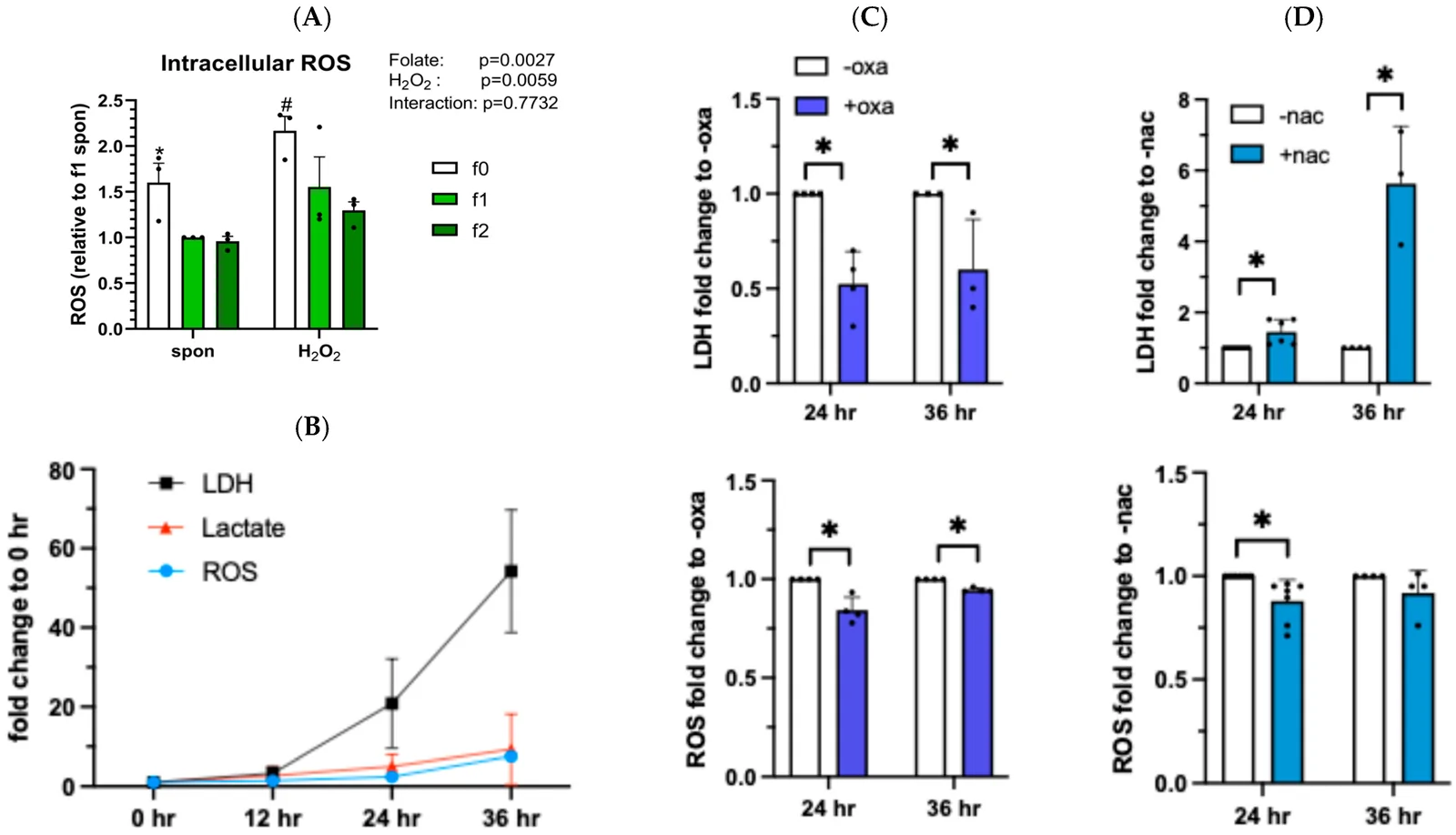
Effects of folate deficiency on oxidative stress, intracellular ROS, and lactate levels in HK-2 cells. (**A**) Intracellular ROS in cells cultured with varying folate concentrations. (**B**) Fold changes in LDH activity, lactate secretion, and ROS levels during 36 hours (hr) of culture under folate-deficient conditions. (**C**) Fold changes in LDH activity and ROS levels following oxamate (20 mM) treatment (+oxa) under folate-deficient conditions. (**D**) Fold changes in LDH activity and ROS levels following N-acetyl-L-cysteine (5 mM) treatment (+nac) under folate-deficient conditions. Data are presented as mean ± SD (*n* = 3–7 independent experiments). * *p* < 0.05, 0.05 < # *p* < 0.10 compared with the corresponding control groups (-oxa or -nac).

Intracellular ROS levels can be increased by oxidative stress induced not only by exogenous hydrogen peroxide but also by folate deficiency (Figure 5A). To further investigate the relationship between intracellular ROS and lactate levels in folate-deficient conditions, changes in LDH activity, lactate levels, and ROS levels were monitored over 36 h of culture (Figure 5B). LDH activity increased earlier during culture under folate-deficient conditions. When LDH activity was inhibited with the LDH inhibitor oxamate, ROS levels were also significantly lower than those observed without LDH inhibition (-oxa) (Figure 5C).

In contrast, when ROS was neutralized with N-acetyl-L-cysteine (+nac), LDH activity remained significantly elevated throughout the 36-h culture period under folate-deficient conditions (Figure 5D). Although, ROS levels decreased after 24 h of nac treatment, the reduction was no longer statistically significant after 36 h, suggesting that additional ROS may have been generated to counteract the ROS-scavenging effect of nac.

Taken together, these findings suggest that folate deficiency initially induces LDH activity and lactate production, which subsequently promotes ROS generation.

### 2.6. Folate Deficiency and Mitochondria Function

**Figure 6:**
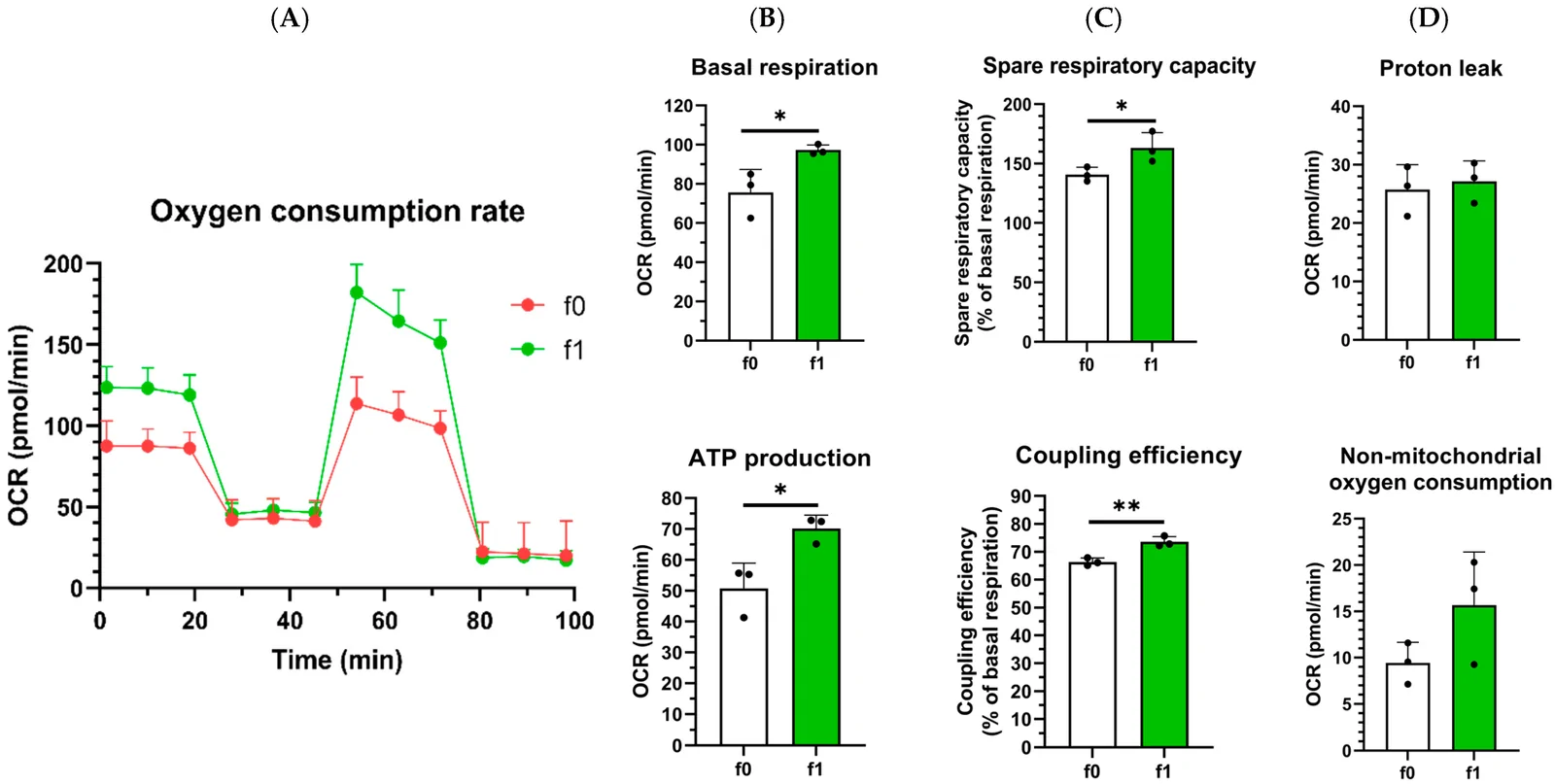
Effect of folate deficiency on mitochondrial function in HK-2 cells. (**A**) Oxygen consumption rates (OCRs) of cells during the Mito-Stress Tests. (**B**) Basal respiration and ATP-linked respiration as an indicator of cellular energy production. (**C**) Spare respiratory capacity, expressed as a percentage of basal respiration, and coupling efficiency as indicators of mitochondrial function. (**D**) Proton leakage and non-mitochondrial oxygen consumption as indicators of mitochondrial respiratory integrity and overall cellular biogenetic status. Cells were cultured in folate-deficient medium (f0; 0 mg/L folate) or folate-sufficient medium (f1; 1 mg/L folate) for 4 days. Data are presented as mean ± SD (*n* = 3 independent experiments). * *p* < 0.05, ** *p* < 0.01 compared with the f1 group, as determined by Student’s *t*-test.

We hypothesized that the elevated mitochondrial ROS (mitoROS) and LDH activity observed in folate-deficient HK-2 cells may reflect impaired mitochondrial function. To investigate this possibility, a mitochondrial stress test performed to assess the mitochondrial respiratory capacity of folate-deficient HK-2 cells. As shown in Figure 6A, the oxygen consumption rate (OCR) in the f0 group remained lower than that in the f1 group throughout the test. Consistent with this finding, folate deficiency significantly reduced basal respiration and ATP production (Figure 6B), as well as spare respiratory capacity and coupling efficiency (Figure 6C). In contrast, proton leakage and non-mitochondrial oxygen consumption were unaffected (Figure 6D).

Collectively, these results demonstrate that folate deficiency impairs mitochondrial respiratory function in HK-2 cells. The lack of changes in proton leakage and non-mitochondrial oxygen consumption suggests that this dysfunction is unlikely to be primarily cause of the increased proton leakage or non-specific oxygen consumption. Instead, they may be related to impaired substrate availability and/or electron transport chain function.

### 2.7. Fructose and LPS Increased Inflammatory Cytokine Secretion and Lactate Production in HK-2 Cells

**Figure 7:**
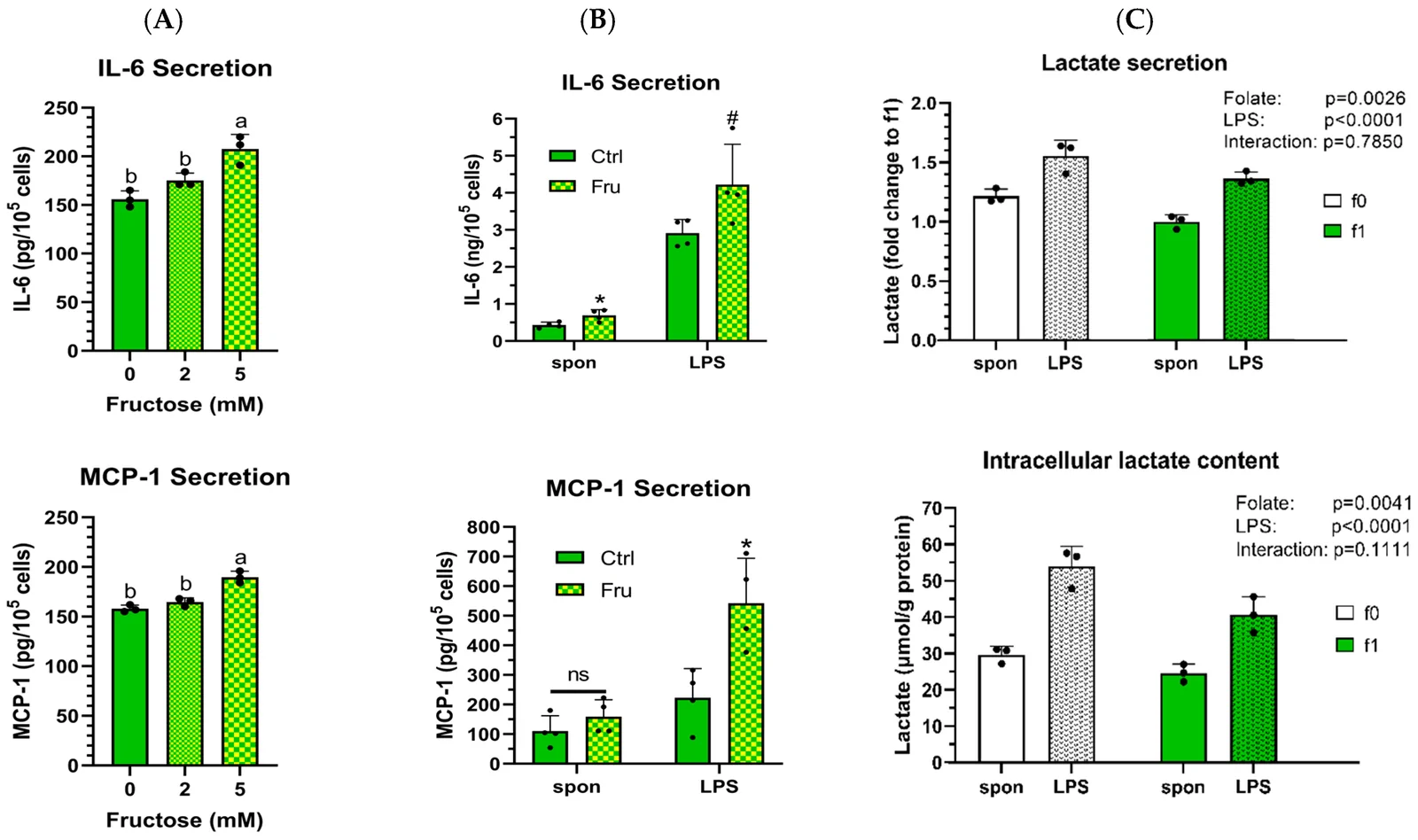
Effects of exogeneous fructose and LPS on IL-6 and MCP-1 secretion and lactate levels in HK-2 cells. (**A**) IL-6 and MCP-1 secretion by cells cultured with varying fructose concentrations. (**B**) IL-6 and MCP-1 secretion by cells cultured with 5 mM fructose following LPS treatment. (**C**) Extracellular lactate secretion and intracellular lactate levels in folate-deficient (f0) or folate-sufficient (f1) HK-2 cells. Cells were cultured in folate-deficient medium (f0; 0 mg/L folate) or folate-sufficient medium (f1; 1 mg/L folate) for 4 days. LPS stimulation (final concentration, 400 ng/mL) was applied during the last 24 h of culture. Data are presented as mean ± SD (*n* = 3–4 independent experiments). * *p* < 0.05, 0.05 < # *p* < 0.10 compared with the corresponding Ctrl group. Different letters indicate significant differences among groups (*p* < 0.05; one-way ANOVA followed by Duncan’s post hoc test).

To investigate whether other dietary factors or inflammatory stimuli affect the secretion of IL-6 and MCP-1 by HK-2 cells, cells were exposed to increasing concentrations of fructose within the range tolerated by the cells. A higher fructose concentration (5 mM) significantly increased the secretion of both IL-6 and MCP-1 (Figure 7A). Furthermore, when HK-2 cells were cultured with 5 mM, LPS further increased the secretion of IL-6 and MCP-1 (Figure 7B).

To determine whether LPS also promote lactate production, HK-2 cells were cultured under folate-deficient or folate-sufficient conditions in the absence or presence of LPS. Both folate deficiency and LPS significantly increased lactate secretion and intracellular lactate levels (Figure 7C).

Taken together, these findings demonstrated that HK-2 cells increase the secretion of the pro-inflammatory cytokines IL-6 and MCP-1 in response to high fructose exposure and LPS stimulation. These results suggest that prolonged exposure to elevated fructose levels or metabolic endotoxemia may contribute to the initiation or progression of kidney injury.

## 3. Discussion

Chronic inflammation is one of the most important features of CKD and places substantial metabolic stress on renal tubular cells [28,29,30,31]. Renal proximal tubules contain an abundance of mitochondria and are critical for the energy-demanding processes involved in the reabsorption of water and solutes. Persistent inflammation signaling can disrupt mitochondrial function, impair oxidative phosphorylation, and increase mitochondrial ROS production [32,33], thereby increasing the susceptibility of kidneys to oxidative stress-induced damage. Therefore, investigating biomarkers of oxidative status may be valuable for the early prediction and prevention of CKD [34].

ROS and inflammation are closely linked through a bidirectional relationship. ROS act as warning signals that trigger immune responses, while activated immune cells produce additional ROS to combat cellular threats. When this cycle becomes dysregulated, excess ROS production can cause tissue damage and contribute to the development and progression of chronic diseases. A previous study showed that cytokine-stimulated endothelial cells increase ROS production, likely through NADH oxidase, and that this increase may be critically involved in the secretion of MCP-1 and IL-6 [35]. ROS can also activate redox-sensitive transcription factors, such as nuclear factor-kappa B (NF-κB), which subsequently upregulates the expression of pro-inflammatory cytokines, thereby perpetuating the inflammatory cascade [36,37]. We demonstrated that the NF-κB signal pathway was activated through phosphorylation of the p65 subunit, facilitating the translocation of NF-κB into the nucleus, where it binds DNA and promotes the transcription of pro-inflammatory cytokines, including IL-6 and MCP-1. This activation was observed with folate deficiency alone and was increased further in combination with LPS. Our findings indicate that nutrient deficiency may contribute to the elevation of pro-inflammatory cytokines.

Early studies showed that cells increase ROS generation during hypoxia and that ROS can upregulate glycolysis under hypoxic conditions. In hepatoma cells, LDH activity was decreased by antioxidants or by overexpression of superoxide dismutase, which eliminates ROS [38,39], suggesting an association between ROS and metabolic shifts. A later study demonstrated a link between LDH and cellular hydrogen peroxide production or oxidative stress in Hela cancer cells, both in vitro and in vivo, suggesting that LDH may amplify ROS in cancer cells in response to oxidative stimuli [40]. Another study showed that treatment with the organic peroxide tert-butyl hydroperoxide (t-BHP), which induces oxidative stress, increased LDH activity in hair-like HEI-OC1 cells [41]. In the current study, we not only demonstrated an association between ROS and lactate production in renal tubular HK-2 cells but also showed that increased lactate production promotes ROS generation. These findings suggest that folate deficiency induces LDH activity and lactate production, which subsequently promote ROS generation.

In this study, we first demonstrated that folate deficiency significantly increases lactate production by enhancing LDH activity, thereby increasing ROS production in HK-2 cells. Some studies have reported that lactate promotes resistance to oxidative stress in neuroblastoma cells or scavenges hydroxyl radicals in several cell types under conditions of elevated labile iron pools [42,43]. However, a recent study showed that lactate can induce oxidative stress through HIF1α stabilization in the mammary glands of dairy cows [44], whereas mitochondrial lactate venting limits oxidative stress, such that pharmacological inhibition of lactate export results in ROS accumulation in the mitochondrial matrix of human embryonic kidney cells (HEK293) [45]. Therefore, the relationship between lactate and ROS may depend on cell type and physiological conditions. Our results demonstrate that elevated lactate levels under folate-deficient conditions promote ROS production in renal tubular HK-2 cells.

ROS are normal byproducts of cellular respiration; however, excessive ROS production can initiate a detrimental feedback loop that exacerbates mitochondrial dysfunction. Mitochondria generate ROS primarily from the electron transport chain, and excess ROS can subsequently damage mitochondrial DNA, lipids, and proteins, further impairing energy production and promoting cell death. Lactate levels and oxygen consumption rate (OCR) are closely related indicators of cellular metabolism. OCR reflects mitochondrial oxidative phosphorylation, whereas lactate is both a product and regulator of glycolysis. Together, these measures provide complementary information for assessing changes in cellular energy metabolism. Exogenous lactate has been shown to increase OCR in cells, suggesting that lactate may enhance mitochondrial respiration and oxidative phosphorylation, thereby potentially increasing ROS production [46]. Nevertheless, further studies are required to establish the complete causal relationship between lactate metabolism, mitochondrial respiration, and ROS generation.

A previous study demonstrated that folate deprivation promotes mitochondrial oxidative damage, including large-scale DNA deletions, cytochrome c oxidase dysfunction, membrane depolarization, and superoxide overproduction in the rat liver [47]. In the present study, we demonstrated that folate deficiency significantly increases oxidative stress at both the cellular and mitochondrial levels in HK-2 cells, which was further exacerbated by lactate supplementation and attenuated by inhibition of lactate production. Furthermore, folate deficiency significantly impaired mitochondrial function and cellular energy production, which may represent a pivotal driver of kidney disease pathogenesis [48]. Therefore, folate may play an important role in maintaining mitochondrial function in the kidney. Given the high metabolic demands of renal tubular cells, folate insufficiency may increase oxidative stress and impair mitochondrial energy production, thereby contributing to renal injury during CKD progression. Although further validation in animal models and clinical studies is warranted, our findings in HK-2 cells suggest that folate deficiency may promote oxidative stress through alterations in lactate metabolism and mitochondrial respiration, providing a potential mechanistic link between folate deficiency and CKD progression.

## 4. Materials and Methods

### 4.1. Cell Culture

HK-2 cells, an established human proximal tubular epithelial cell line derived from the normal kidney cortex of an adult male, were used to investigate relationships among folate, inflammation, and oxidative stress during the progression of nephritis. The HK-2 cell line is commercially available from the American Type Culture Collection (ATCC; Manassas, VA, USA). However, the cells used in this study were obtain from the collaborating laboratory of Dr. Tien Hsu, Graduate Institute of Biomedical Sciences, China Medical University, Taichung, Taiwan, rather than directly from ATTC. Before expansion, HK-2 cells were maintained in complete RPMI 1640 (Thermo Fisher, Taipei, Taiwan) supplemented with 10% heat-inactivated FBS (Biological Industries, Cromwell, CT, USA) and a penicillin-streptomycin-amphotericin (PSA) antibiotic cocktail (Gibco, Waltham, MA, USA) at 37 °C in a humidified atmosphere containing 5% CO_2_. The cells were not routinely screened for mycoplasma contamination during these experiments.

Specifically, HK-2 cells (passages 5–10) were cultured in folate-free RPMI 1640 supplemented with 10% heat-inactivated FBS and a PSA antibiotic cocktail at 37 °C in a humidified atmosphere containing 5% CO_2_. Following cell attachment, cells were cultured for 4 days in folate-deficient medium (f0 (0 mg/L folate) or f0.25 (0.25 mg/L folate)), folate-sufficient medium (f1; 1 mg/L folate), or folate-supplemented medium (f2; 2 mg/L folate or f5; 5 mg/L folate). Intracellular folate contents were measured to validate the folate-deficiency cell model. (Supplementary Figure S1).

### 4.2. Determination of Cytokine and Chemokine Secretion Levels

Secretion levels of cytokines and chemokine, including IL-6, MCP-1, and TGF-β1, were determined using commercial ELISA kits (Invitrogen, Carlsbad, CA, USA; BioLegend, San Diego, CA, USA). Briefly, cell culture supernatants from cultured HK-2 cells were collected and appropriately diluted prior to ELISA. The assays were performed according to the manufacturers’ protocols, including coating the capture antibodies, incubation with samples and standards, and incubation with detection antibodies, followed by HRP-conjugate binding and substrate development for colorimetric detection. Absorbance was measured at 450 nm, and cytokine concentrations were calculated by interpolation from the corresponding standard curves.

### 4.3. NF-kB Pathway Activation Assay

NF-κB pathway activation was determined by Western blotting. Cell lysates were prepared using RIPA buffer, and protein concentrations were determined using the BCA assay. Equal amounts of protein were separated by SDS-PAGE and transferred onto 0.45-μm PVDF membranes (Amersham, Buckinghamshire, UK). The membranes were blocked with 5% non-fat milk in TBST and incubated with primary antibodies against phospho-NF-κB p65 (Ser536) (Cell Signaling Technology, Danvers, MA, USA; 1:1000), NF-κB p65 (Cell Signaling Technology; 1:1000) and GAPDH (Proteintech, Rosemont, IL, USA; 1:5000), followed by incubation with the corresponding HRP-conjugated secondary antibodies. Protein bands were visualized using T-Pro Lumilong Plus Chemiluminescent Substrate (T-Pro Biotechnology, New Taipei County, Taiwan).

### 4.4. Measurements of Lactate and Lactate Dehydrogenase Activity

#### 4.4.1. Lactate Secretion and Intracellular Lactate

To determine the intra- and extracellular lactate contents, HK-2 cells and the culture supernatants were collected and analyzed using a colorimetric lactate assay kit (Abcam, Waltham, MA, USA). Both intracellular and extracellular lactate levels were normalized to total protein content, as determined by the BCA assay (Thermo Fisher).

#### 4.4.2. Lactate Dehydrogenase Activity Assay

To determine LDH activity, HK-2 cells were collected and analyzed using a colorimetric LDH activity kit (Abcam). LDH activity was determined based on the average reaction rate over 4 min at 37 °C and normalized to total protein content.

### 4.5. Pharmacological Inhibitor and Exogenous Factor Experiments

#### 4.5.1. Inhibition of Lactate Dehydrogenase and Exogenous Lactate Supplementation

To determine the effect of LDH inhibition and exogenous lactate supplementation on cytokine secretion and ROS accumulation, HK-2 cells were treated with 20 mM sodium oxamate (Sigma, Singapore, Singapore) in the presence or absence of 10 mM ethyl L-lactate (Sigma) for 4 days [49].

#### 4.5.2. ROS Neutralization

To determine the involvement of ROS, HK-2 cells were treated with 5 mM N-acetyl-L- cysteine (NAC; Yu-Ho Chemical Co., Ltd., Hangzhou, China) under the indicated folate conditions.

#### 4.5.3. Measurement of Reactive Oxygen Species

Intracellular ROS levels were determined using 2′,7′-dichlorodihydrofluorescein diacetate (DCFH-DA; Sigma-Aldrich, Singapore, Singapore). HK-2 cells were incubated with 10 μM DCFH-DA in serum-free medium at 37 °C for 30 min in the dark. After washing twice with PBS, fluorescence intensity was measured at excitation and emission wavelengths of 485 and 520 nm, respectively.

Mitochondrial ROS were determined using MitoSOX™ Red Mitochondrial Superoxide Indicator (MedChemExpress, Monmouth Junction, NJ, USA). HK-2 cells were incubated with 5 μM MitoSOX Red at 37 °C for 10 min in the dark. After washing with PBS, cells were immediately imaged using a fluorescence microscope (Leica Microsystems, Wetzlar, Germany). Three random fields were captured at 200× magnification, and the mean fluorescence intensity was quantified using Leica Application Suite X (version 3.7.4.23463).

#### 4.5.4. LPS Stimulation

To determine the cytokine secretion profiles upon LPS stimulation, HK-2 cells were treated with LPS at a final concentration of 400 ng/mL (Sigma) for 24 h after either 3 days of folate-deficient culture [50,51,52] or 24 h of fructose treatment (Archer Daniels Midland Company, Chicago, IL, USA). HK-2 cell viability was not significantly affected by the treatment conditions (Supplementary Figure S2). The same treatment conditions were used to determine the effects of LPS on lactate accumulation and secretion.

### 4.6. Measurement of Oxygen Consumption Rate

#### 4.6.1. Assay Preparation

To assess mitochondrial function under folate-deficient conditions, HK-2 cells were seeded onto Seahorse XF24 microplates and allowed to adhere for 18 h prior to the assay. On the day of the assay, the culture medium was replaced with Seahorse XF RPMI Medium supplemented with 11 mM glucose and 2 mM L-glutamine (pH 7.4). The plates were equilibrated in a non-CO_2_ incubator at 37 °C for 1 h prior to measurement.

#### 4.6.2. Mito-Stress Test

Oxygen consumption rates (OCRs) were measured using a Seahorse XFe24 Analyzer (Agilent, Singapore, Singapore) and the XF Cell Mito Stress Test Kit (Agilent) under basal conditions and following sequential injections of oligomycin (1 μM), FCCP (1 μM), and a combination of rotenone and antimycin A (0.5 μM each). These compounds inhibit ATP synthase, uncouple oxidative phosphorylation from the electron transport chain, and inhibit mitochondrial complexes I and III, respectively. OCR measurements were used to calculate basal respiration, ATP-linked respiration, maximal respiration, and spare respiratory capacity according to the manufacturer’s instructions.

### 4.7. Statistical Analysis

Data are presented as mean ± SD. Student’s *t*-test was used for comparisons between two groups, whereas one-way or two-way ANOVA followed by Duncan’s post hoc test was used for multiple-group comparisons. Statistical significance was set at *p* < 0.05.

## 5. Conclusions

**Figure 8:**
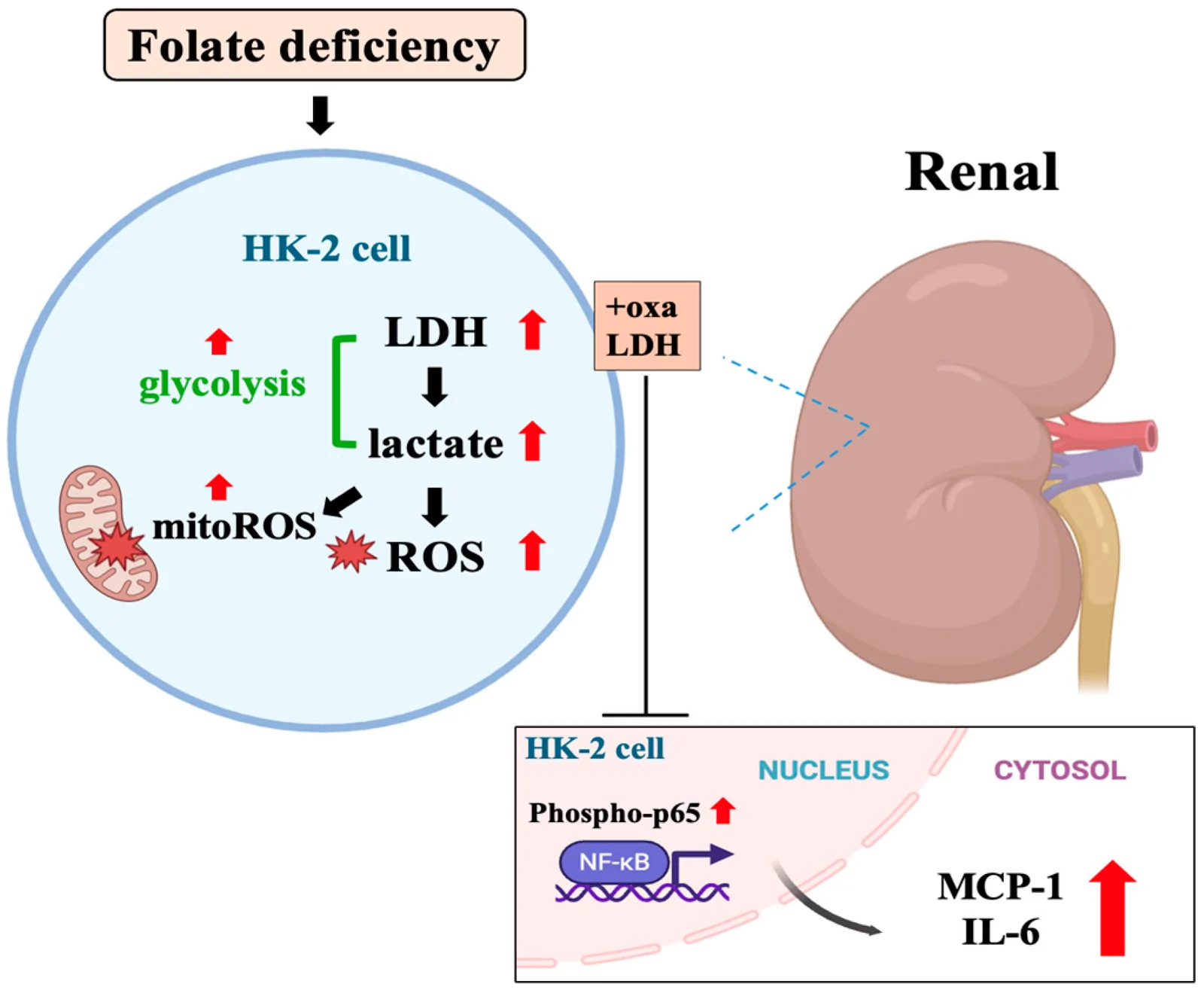
Schematic diagram illustrating the effects of folate deficiency on metabolic alterations in human proximal epithelial HK-2 cells. Folate deficiency promotes secretion of the pro-inflammatory cytokines IL-6 and MCP-1 through activation of the NF-κB pathway and increases lactate production by enhancing lactate dehydrogenase activity. Elevated lactate levels further promote the secretion of IL-6, MCP-1, and TGF-β1. Increased lactate levels also enhance cellular and mitochondrial oxidative stress and contribute to mitochondria dysfunction, particularly under folate-deficient conditions. The increased oxidative stress and inflammatory and fibrotic cytokine production induced by folate deficiency in renal tubular cells may contribute to the progression of CKD. Black arrows indicate the proposed pathway, while red upward arrows show increases in these factors.

As summarized in Figure 8, folate deficiency promotes inflammation, dysregulated lactate metabolism, oxidative stress, and mitochondrial dysfunction in human renal tubular epithelial cells. Elevated lactate levels further enhanced pro-inflammatory and profibrotic signaling, NF-kB activation, and ROS generation, suggesting that lactate accumulation may serve as a metabolic link between folate deficiency and renal injury. High fructose exposure also exacerbated inflammatory responses, particularly under LPS stimulation. Collectively, these findings suggest that folate deficiency and dysregulated lactate metabolism may contribute to the initiation and progression of CKD by linking metabolic dysfunction with inflammation and oxidative stress, highlighting folate status and lactate metabolism as potential targets for further investigation in the prevention of diet-associated renal injury.

## Data Availability

The master’s dissertation supporting this study is available in the National Taiwan University Library. Specific underlying research data can be obtained from the corresponding author upon reasonable request.

## Supplementary Materials

The following supporting information can be downloaded at: https://www.mdpi.com/article/10.3390/ijms27198846/s1.

## Abbreviations

The following abbreviations are used in this manuscript:

CKD	Chronic kidney disease
LDH	Lactate dehydrogenase
MCP-1	Monocyte chemoattractant protein
mito	mitochondria
nac	N-acetyl-L-cysteine, a ROS neutralizer
NF-kB	Nuclear factor kappa B
oxa	Oxamate, an LDH inhibitor
ROS	Reactive oxygen species
TGF-β	Transforming growth factor beta
