# Supplementary material for: Folate Deficiency Drives Metabolic Dysfunction, Oxidative Stress, and Inflammation in HK-2 Renal Tubular Cells
Source: Int J Mol Sci. 2026 Oct 3;27(19):8846. doi: 10.3390/ijms27198846 (PMC13665185; doi:10.3390/ijms27198846)
Supplement: Supplementary file 1 [file ijms-27-08846-s001.zip › ijms-4564729-supplementary.pdf]

## supplementary Figure

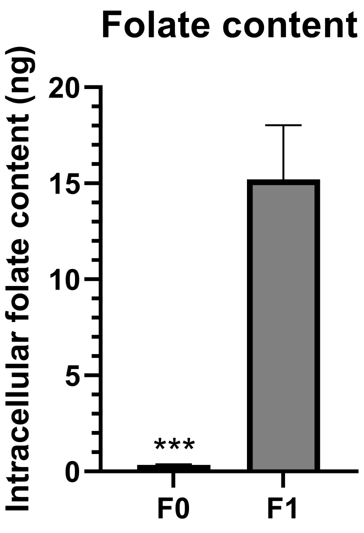


Supplementary Figure S1 Intracellular folate content of HK-2 cells after 4-day folate-deficient culture.
Data were mean±SD, n=5 independent experiments. ***$p<0.001$, compared with F1 (folate-sufficient, 1 mg/L) group by unpaired Student's t test.

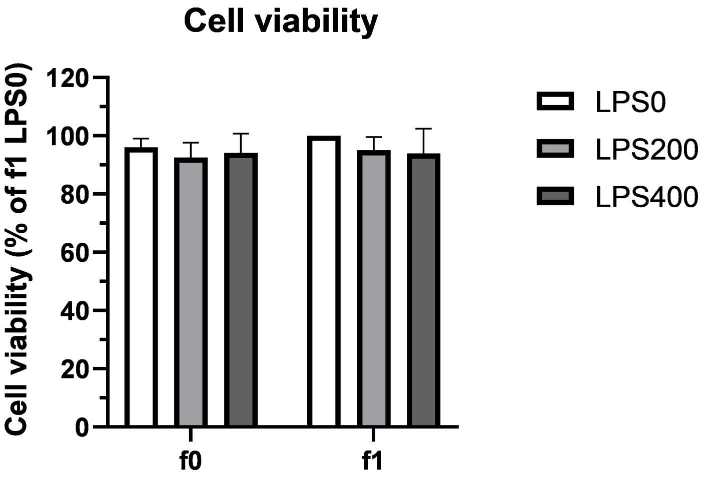


Supplementary Figure S2 Cell viability of HK-2 cells after 4-day folate-deficient culture, with LPS stimulation in the last 24 hours. Data were mean±SD, n=3 independent experiments.
